# Nek2B activates the wnt pathway and promotes triple-negative breast cancer chemothezrapy-resistance by stabilizing β-catenin

**DOI:** 10.1186/s13046-019-1231-y

**Published:** 2019-06-07

**Authors:** Honghong Shen, Wenpeng Yan, Jinyang Yuan, Ziyue Wang, Chen Wang

**Affiliations:** 1 Department of pathology, The Second Clinical Medical College, Shanxi Medical University, 382 Wuyi Road, Taiyuan, 030001 People’s Republic of China; 2 Department of general surgery, The Second Clinical Medical College, Shanxi Medical University, 382 Wuyi Road, Taiyuan, 030001 People’s Republic of China

**Keywords:** Nek2B, β-Catenin, Wnt signaling pathway, Triple-negative breast cancer, Chemotherapy resistance

## Abstract

**Background:**

The chemotherapy-resistance of triple-negative breast cancer (TNBC) remains a major challenge. The Nek2B kinase and β-catenin serve as crucial regulators of mitotic processes. The aim of this study was to test the correlation between Nek2B and TNBC chemotherapy sensitivity, and to determine the regulation of Nek2B on β-catenin and wnt/β-catenin signal pathway.

**Methods:**

Gene Expression Omnibus(GEO) databases were used to gather gene exprsssion data of TNBC patients who undergoing chemotherapy. The co-expression of Nek2B and β-catenin in TNBC surgical sections and cells were analysed by immunohistochemistry, Q-RT-PCR, Western-blot and immunofluorescent staining. The impact of the expression of Nek2B and β-catenin in prognosis was also assessed using the Kaplan-Meier curves. CCK8 assay was used to detect the IC50 value of TNBC cell line. The endogenous binding capacity of Nek2B and β-catenin and phosphorylation of β-catenin by Nek2B were detected using co-immunoprecipitation (CO-IP). Chromatin immune-precipitation (ChIP) analysis and Luciferase Assays were used to evaluate the binding ability of the Nek2B, β-catenin and TCF4 complex with LEF-1 promoter. Nek2B-siRNA and Nek2B plasmid were injected into nude mice, and tumorigenesis was monitored.

**Results:**

We found that overexpression of Nek2B and β-catenin in TNBC samples, was associated with patients poor prognosis. Patients with positive Nek2B expression were less sensitive to paclitaxel-containing neoadjuvant chemotherapy. Interestingly, in a panel of established TNBC cell line, Nek2B and β-catenin were highly expressed in cells exhibiting paclitaxel resistance. Our data also suggest that β-catenin binded to and was phosphorylated by Nek2B, and was in a complex with TCF4. Nek2B mainly regulates the expression of β-catenin in TNBC nucleus. Nek2B, β-catenin and TCF4 can be binded with the WRE functional area of LEF-1 promoter. Nek2B can activite wnt signaling pathway and wnt downstream target genes. The tumors treated by Nek2B siRNA associated with paclitaxel were the smallest in nude mouse, and Nek2B can regulate the expression of β-catenin and wnt downstream target genes in vivo.

**Conclusion:**

Our study suggested that Nek2B can bind to β-catenin and the co-expression correlated with TNBC patients poor prognosis. It appears that Nek2B and β-catenin might synergize to promote chemotherapy resistance.

## Background

Approximately 15% of breast cancers are classified as triple-negative breast cancer (TNBC), lacking expression of the estrogen receptor and the progesterone receptor, and which is characterized by absence or low expression or no amplification on the gene level, of the human epidermal growth factor receptor 2 [1, 2]. Treatment of TNBC, which often presents with a more aggressive phenotype, is more difficult due to the paucity of potential target molecules. Therefore, there is a critical need to enhance current systemic treatments and identify new targets for the treatment of TNBC [3].

Because the centrosome cycle is regulated by protein phosphorylation and given the importance of mitotic and centrosomal kinases, they are attractive targets for anti-mitotic anticancer drugs [4]. NIMA-related kinase 2 (Nek2), which has at least three spliced isomers: Nek2A, Nek2B and Nek2C, is such a target. Nek2B is the only Nek2 isomer that runs through the whole process of mitosis [5]. It can regulate centrosome aggregation and separation by regulating the mitotic centrosome separation through reversible phosphorylation of its substrates [6, 7]. High levels of Nek2B are linked to a poor disease-free survival (DFS) and overall survival (OS) in TNBC and other cancers [8]. Nek2 depletion affected the kinetochores-microtubule attachment, inducing a spindle checkpoint imbalance and abnormal clustering of kinetochore components, resulting in increased sensitivity of cells to the microtubule targeting anticancer drug paclitaxel [9]. In spite of the increasing evidence of the importance of Nek2B in cancer development, its role in cancer is still far from clear.

The wnt signaling pathway is an important extracellular pathway comprises two different intracellular signaling pathways: the β-catenin-dependent pathway (known as the canonical Wnt pathway) and the β-catenin-independent pathway (known as the noncanonical Wnt pathway). Compared with other signaling pathways, the canonical Wnt/β-catenin pathway is highly evolutionary conserved and mainly involves β-catenin [10]. β-catenin is a well-studied protein with known functions in the nucleus in a complex with the transcriptional coactivator T-cell factor/lymphocyte enhancer factor-1 (TCF/LEF-1) [11]. Similar to Nek2, β-catenin localizes to centrosomes throughout the cell cycle, and β-catenin localization to interphase centrosomes is mediated by the linker proteins [12]. β-catenin was also identified as a substrate for Nek2 kinase in vivo and in vitro [13]. Nek2 activity is regulated during the cell cycle and peaks at the G2/M boundary when β-catenin localization to centrosomes. The peak of Nek2 activity at the G2/M boundary coincides with a linker-protein independent increase of β-catenin at centrosomes [14]. However, studies concerning the expression of Nek2B and β-catenin in human TNBC have not been reported previously.

Our goal in this study is to identify a reliable, clinically useful prognostic gene-expression signature that offers novel clinical opportunities for predicting drug resistance and developing novel treatments in TNBC. We determined the effects of Nek2B-overexpression on drug resistance and explored the binding abilitity of Nek2B and β-catenin in TNBC cells.

## Materials and methods

### Patients and tissue specimens

One hundred and seventy-four TNBC breast cancer samples were obtained from 1114 breast cancer patients at the Second Clinical Medical College, Shanxi Medical University, China, during January 1, 2007 and December 31, 2012. Patients who met the following criteria were eligible for this study: (1) patients with invasive TNBC, (2) patients who have completed follow-up date during the study period.

### Immunohistochemistry

Immunohistochemical analyses were performed on formalin-fixed, paraffin-embedded sections of surgical specimen as described previously [15]. The primary antibody rabbit monoclonal anti-human Nek2B antibody (ab227958, Abcam, Cambridge, MA, USA) at 1:250 dilution or mouse monoclonal anti-human β-catenin antibody (ab32572, Abcam) at 1:200 dilution was incubated overnight at 4 °C. For scoring Nek2B and β-catenin expression, the intensity (intensity score: 0 = negative, 1 = weak, 2 = moderate, and 3 = strong) and percentage of the total cell population (proportion score: 0, 0%; 1, <25%; 2, 25–49%; 3, 50–74%; 4, ≥ 75%) were evaluated for each case. The expression was regarded as negative (intensity multiply proportion scores 0–1) or positive (intensity multiply proportion scores ≥2) using high-powered (× 200) microscopy.

### Cell lines and culture conditions

Human TNBC cell lines Hs578T, BT20, MDA-MB-231 and MDA-MB-468 were chosen. Cells were cultured in DMEM medium (Gibco) or RPMI 1640(Gibco), 10% fetal bovine serum (FBS)(Gibco) and 1% penicillin/streptomycin (Life Technologies Inc), under a humidified atmosphere without CO_2_ at 37 °C.

### CCK8 assay

Cell proliferation viability was measured with a Cell Counting Kit-8 (CCK8, Beyotime) following the manufacturer’s instructions. The absorbance was measured at 450 nm on a microplate reader. The OD value was checked every 24 h. Each experiment was performed in triplicate.

### Reverse transcription quantitative real-time polymerase chain reaction (Q-RT-PCR)

Total RNA was isolated from cells, and qPCR was performed as described previously [15]. Target gene expression values were normalized to Actin. The primers used for qPCR are as follows:

*Actin*-Fwd:5′-AGCGAGCATCCCCCAAAGTT-3′

*Actin*-Rev:5′-GGGCACGAAGGCTCATCATT-3′.

*Nek2B*-Fwd:5′-CTAGCTAGCTAGCCGTCACGGGTCGAG-3′.

*Nek2B*-Rev:5′-CGGGATCCTTAGAATTTGCTCCATTCATTCC-3′.

*Β-catenin*-Fwd:5′-ACTTGCCACACGTGCAATTC-3′.

*Β-catenin*-Rev:5′-ATGGTGCGTACAATGGCAGA-3′.

### Western-blot

The total protein was extracted from each sample using RIPA buffer containing protease inhibitors. Total cell lysates containing 10 μg of protein were electrophoresed on a SDS-PAGE and then transferred to PVDF membranes (Millipore, MA, USA). The sample was blocked with 5% fat-free milk for 1 h, and incubated with primary antibodies for 1 h and with secondary antibodies for 1 h. The primary antibodies, including anti-Nek2B (ab227958; Abcam) at 1:2000 and anti-β-catenin (ab32572; Abcam) at 1:5000 were used in the present study. The blots were visualized using ECL assay.

### Immunofluorescence

Immunofluorescence was performed as described previously [15]. The two set mixed primary antibody including anti-rabbit monoclonal Nek2B antibody (200ul, 1:500) and anti-mouse monoclonal β-catenin antibody (200ul, 1:250). Cells were mounted with DAPI away from light. The signal of Alexa Fluor 488 was visualized as green (Nek2B) and Alexa Fluor 594 as red (β-catenin).

### Nuclear and cytoplasmic extraction

Nuclear and cytoplasmic fractions were isolated using NE-PER Nuclear and Cytoplasmic Extraction Reagents (Thermo Fisher Scientific, Waltham, MA) according to the instructions provided by the manufacturer.

### Immune protein coprecipitation (CO-IP)

Cell lysates were incubated for 4 h at 4 °C with the indicated antibodies and protein A/G beads (Thermo Fisher Scientific, Waltham, MA) or with anti-FLAG (Sigma-Aldrich, St. Louis, MO) or anti-HA (Sigma-Aldrich, St. Louis, MO) agarose gels for FLAG-OR HA-tagged protein pull-down. After incubation, beads were washed with low salt lysis buffer, boiled with 4× SDS loading buffer. IB analysis was developed using the Western HRP Chemiluminescence Substrates (Millipore, Burlington, MA) .

### Chromatin immunoprecipitation (ChIP)

ChIP assay was performed according to instruction (Millipore). ChIP assays were performed using primers spanning binding sites in the LEF-1 promoter. 20 μl magnetic AG-beads (Invitrogen) were used for each ChIP. DNA was purified using DNA Purification Buffer plus DNA purification magnetic beads, as recommended (Invitrogen).

### Luciferase assays

TNBC cells were transfected with pGL4-LEF-1-prom-Luc reporters along with plasmids using the Lipofectamie 2000(Invitrogen, USA) as indicated in the figures. After 48 h of transfection, cell lysates were subjected to luciferase assays according to the Dual-Luciferase Reporter Assay protocol (Promega, Madison, WI). Each experiment was repeated in triplicates.

### Invasion assays

Transwell migration assays were performed using a transwell membrane (8-μm pore size, 6.5-mm diameter; Corning Incorporated, Corning, NY, USA) in a 24-well plate according to the manufacturer’s instructions. The cells were placed in incubators at 37 °C for different time periods according to preliminary experiments. The upper surface cells were removed using cotton swabs, those on the lower surface were fixed with 4% paraformaldehyde in PBS, stained with 0.1% crystal violet and counted.

### In vivo experiments

Female BALB/c nude mice of 4–6 weeks old and body weight of 20 ± 2 g were housed in specific pathogenfree (SPF) conditions. All the rats were administered as described previously [16]. All the rats were sacrificed after 30 days and the masses were resected.

### Follow-up study

The primary endpoints were disease-free survival (DFS) and overall survival (OS). DFS was calculated as time from surgery to locoregional recurrence, distant metastasis, or death. OS was determined as the time from surgery until the date of death or was censored at the date of last follow-up.

### Statistical analysis

The statistical significance between Nek2B and β-catenin expression and patients’ clinicopathological status was tested using chi square (χ2) test. The correlation of Nek2B and β-catenin expression with patient prognosis was determined by Kaplan-Meier method. A two-sided *p*-value of< 0.05 was considered significant. All in vitro experiments were performed in triplicate. Data are represented as mean ± S.E. All analyses were performed using SPSS 22.0.

## Results

### Nek2 was listed as an TNBC chemotherapy-resistant candidate biomarker by integrated analysis

**Fig. 1:**
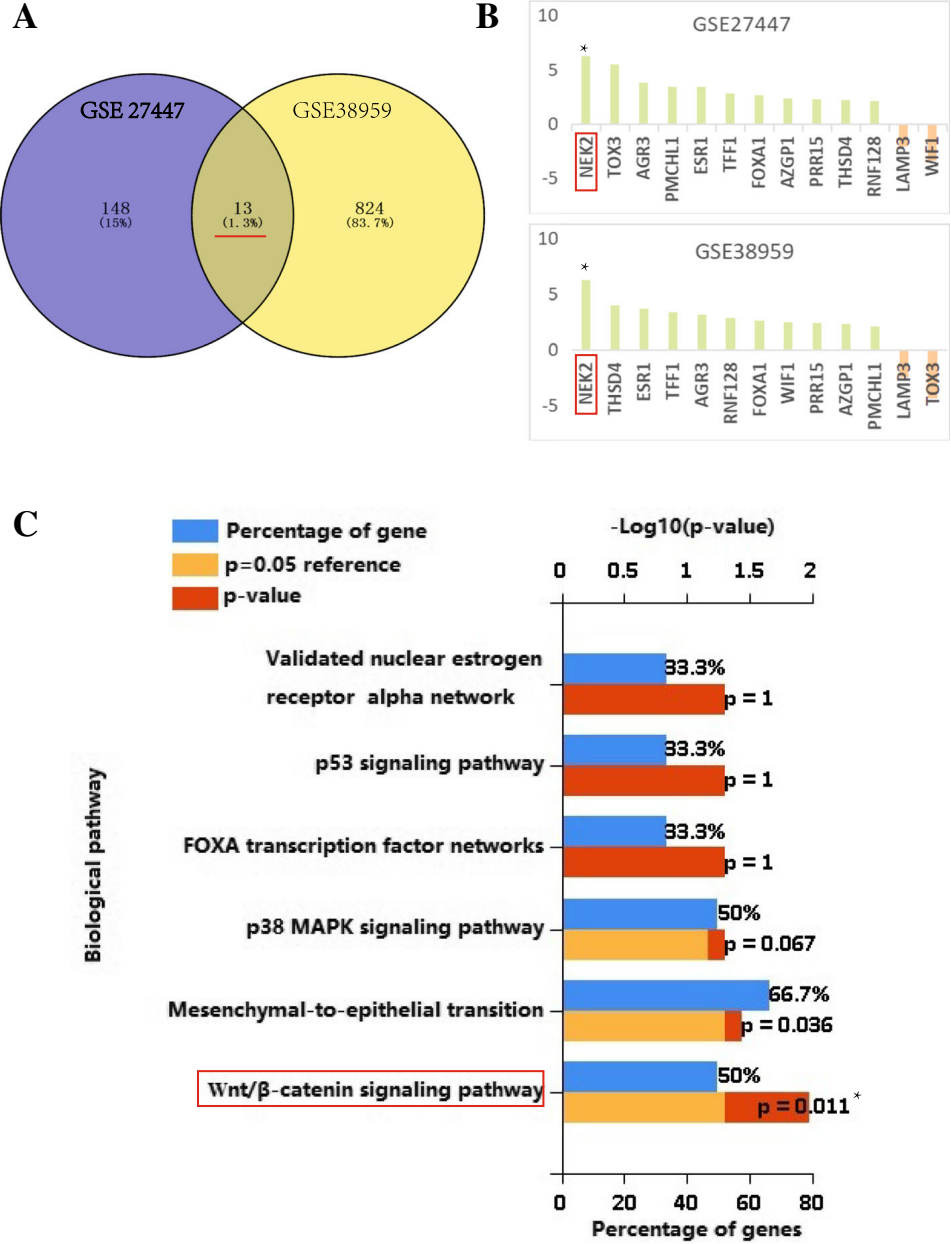
(**a**) Venn diagram of the overlaps between GSE27447 DEGs and GSE38959. DEGs. There were 13 common differential genes between the two circles; (**b**) Relative expression of 13 common differential genes in GSE27447 DEGs and GSE38959 DEGs; (**c**) Enrichment analysis of the KEGG pathway. *P<0.05; **P<0.01

Related gene data set GSE27447 and GSE38959 of TNBC patients who undergoing chemotherapy was downloaded from GEO database and divided into chemotherapy-sensitive group (no local recurrence) and chemotherapy-resistance group (local recurrence). Thirteen differentially expressed genes related to the chemotherapy drug resistance were found using GEO2R analysis tools (Fig. 1a), of which 11 upregulation and 2 downregulation. Nek2 is the most obvious gene of upregulation genes (*P* < 0.05) (Fig. 1b). Gene Ontology (GO) enrichment analysis and Kyoto Encyclopedia of Genes and Genomes (KEGG) pathway enrichment analysis were conducted respectively through DAVID database. GO enrichment analysis showed that differentially expressed genes were significantly enriched in cell cycle, stem cell differentiation, cell apoptosis and other biological processes. The enrichment analysis of KEGG pathway showed that Nek2 was involved in wnt/β-catenin signal pathway (Fig. 1c).

### The expression of Nek2B and β-catenin by immunohistochemistry

**Fig. 2:**
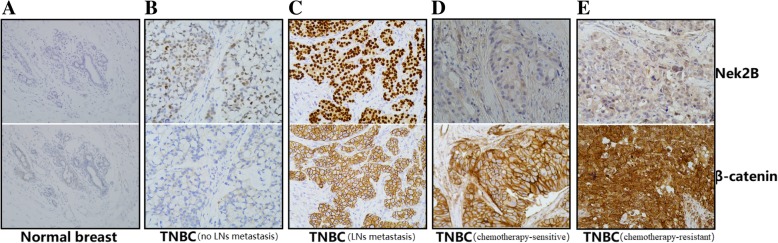
Serial slices with immunohistochemical analysis of Nek2B and β-catenin expression in TNBC specimens: Nek2B (**a up**) and β-catenin (**a down**) (Normal breast) (200×); Nek2B (**b up**) and β-catenin (**b down**) (TNBC (no LNs metastasis) (200×); Nek2B (**c up**) and β-catenin (**c down**) (TNBC (LNs metastasis) (200×); Nek2B (**d up**) and β-catenin (**d down**) (TNBC (chemotherapy-sensitive) (200×); Nek2B (**e up**) and β-catenin (**e down**) (TNBC (chemotherapy-resistant) (200×)

We included in the analysis 174 women with TNBC at the Second Clinical Medical College, Shanxi Medical University, China, during 2007–2012. The Nek2B protein is expressed in cytoplasm and nuclear (Fig. 2 up). The β-catenin protein is mainly expressed in membrane (Fig. 2 down). Low or no expression of Nek2B and β-catenin in normal breast tissue (Fig. 2a);The expression intensity of Nek2B and β-catenin in TNBC tissues with lymph nodes (LNs) metastasis (Fig. 2c) was higher than that without LNs metastasis (Fig. 2b), while it was higher in chemotherapy-resistant tissues (Fig. 2e) than in chemotherapy-sensitive tissues (Fig. 2d).

### The expression of Nek2B and β-catenin with clinical-pathological and prognosis

**Fig. 3:**
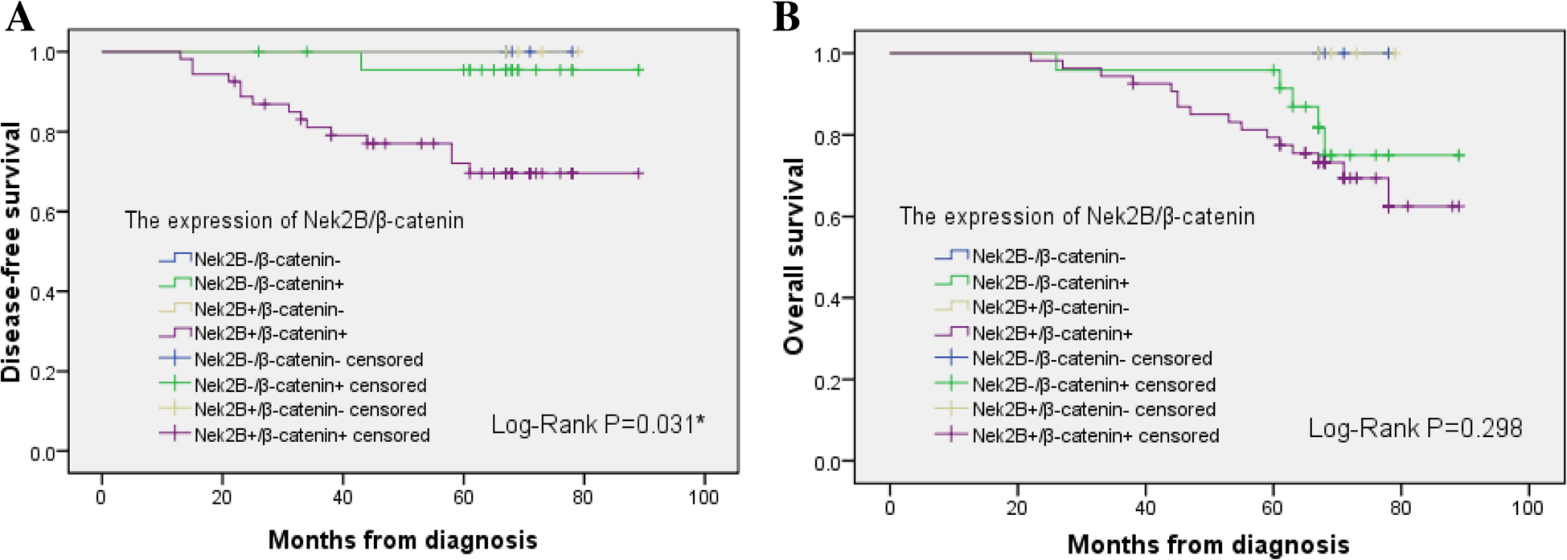
Kaplan-Meier curves for impact of the expression of Nek2B/β-catenin on disease-free survival (**a**) and overall survival (**b**). Log-rank *P* values are shown above

As shown in Table 1, the age of the patients ranged from 36 to 80 years with a mean age and median age of 53.0 years and 51.5 years, respectively. In specimens from patients with TNBC, Nek2B was expressed in 116 cases (66.7%), β-catenin was expressed in 150 cases (86.2%). The number of patients with positive Nek2B expression was significantly higher in the high tumor grade(G3) group (*P*<0.0001), LNs metastasis group (*P* = 0.003), and high Ki67 index group (*P*<0.0001). Nek2B tended to be higher in β-catenin positive cases (72.0%). The number of patients with positive β-catenin expression was significantly higher in the high Ki67 index group (*P* = 0.003). As shown in Fig. 3, the Kaplan-Meier curves showed that patients with Nek2B+/β-catenin+ expression have lower DFS rates compared with other groups(*P* = 0.031).

**Table 1:** The expression of Nek2B and β-catenin with clinicopathological features in TNBC patients

Factors		Nek2B *P*				β-catenin *P*			
		Positive		negative		Positive		negative	
		N	%	N	%	N	%	N	%
All	174	116	66.7	58	33.3	150	86.2	24	13.8
Age					0.824				0.197
≤ 50	110	74	67.3	36	32.7	92	83.6	18	16.4
>50	64	42	65.6	22	34.4	58	90.6	6	9.4
Tumor size (cm)					0.033^a^				0.975
≤ 2	26	14	53.8	12	46.2	24	92.3	2	7.7
>2; ≤ 5	96	72	75.0	24	25.0	82	85.4	14	14.6
>5	52	30	57.7	22	42.3	44	84.6	8	15.4
Tumor grade					< 0.0001^a^				0.593
G1-G2	64	24	37.5	40	62.5	54	84.4	10	15.6
G3	110	92	83.6	18	16.4	96	87.3	14	12.7
LNs metastasis					0.003^a^				0.191
No	80	44	55.0	36	45.0	66	82.5	14	17.5
Yes	94	72	76.6	22	23.4	84	89.4	10	10.6
Ki67					< 0.0001*				0.003^a^
<20%	82	42	51.2	40	48.8	64	78.0	18	22.0
≥ 20%	92	74	80.4	18	19.6	86	93.5	6	6.5
β-catenin					0.001^a^				
Negative	24	8	33.3	16	66.7	–		–	
Positive	150	108	72.0	42	28.0	–		–	
Radiotherapy					0.782				0.814
No	142	94	66.2	48	33.8	122	85.9	20	14.1
Yes	32	22	68.8	10	31.3	28	87.5	4	12.5
Chemotherapy					0.646				0.897
No	56	36	64.3	20	35.7	48	85.7	8	14.3
Yes	118	80	67.8	38	32.2	102	86.4	16	13.6

*LNs* Lymph nodes; ^a^Difference was statistically significant

### The expression of Nek2B with chemotherapy resistance

To explore the relationship between Nek2B expression and paclitaxel resistance, we selected 43 patients diagnosed with TNBC by needle core biopsy, all of whom received 2–3 cycles of neoadjuvant chemotherapy. pCR (pathological complete response) was achieved in 1 patient, PR (partial response) was achieved in 14 patients, SD (stable condition) was achieved in 10 patients, and PD (disease progression) was achieved in 18 patients. Therefore, our research group detected the expression of Nek2B mRNA in 42 postoperative cancer tissues. As shown in Table 2, only 26.7% of patients in the Nek2B positive expression group achieved PR, while up to 53.3% of patients achieved PD(*P* = 0.003). It can be seen that patients with positive Nek2B expression developed drug resistance to paclitaxel-containing neoadjuvant chemotherapy.

**Table 2:** The expression of Nek2B with chemotherapy sensitivity of TNBC patients

	PR	SD	PD	χ^2^	*P* value
Nek2B				13.388	0.003^a^
Negative	6 (50.0%)	4 (33.3%)	2 (16.7%)		
Positive	8 (26.7%)	6 (20.0%)	16 (53.3%)		

*PR* partial response, *SD* stable condition, *PD* disease progression; ^a^Difference was statistically significant

**Fig. 4:**
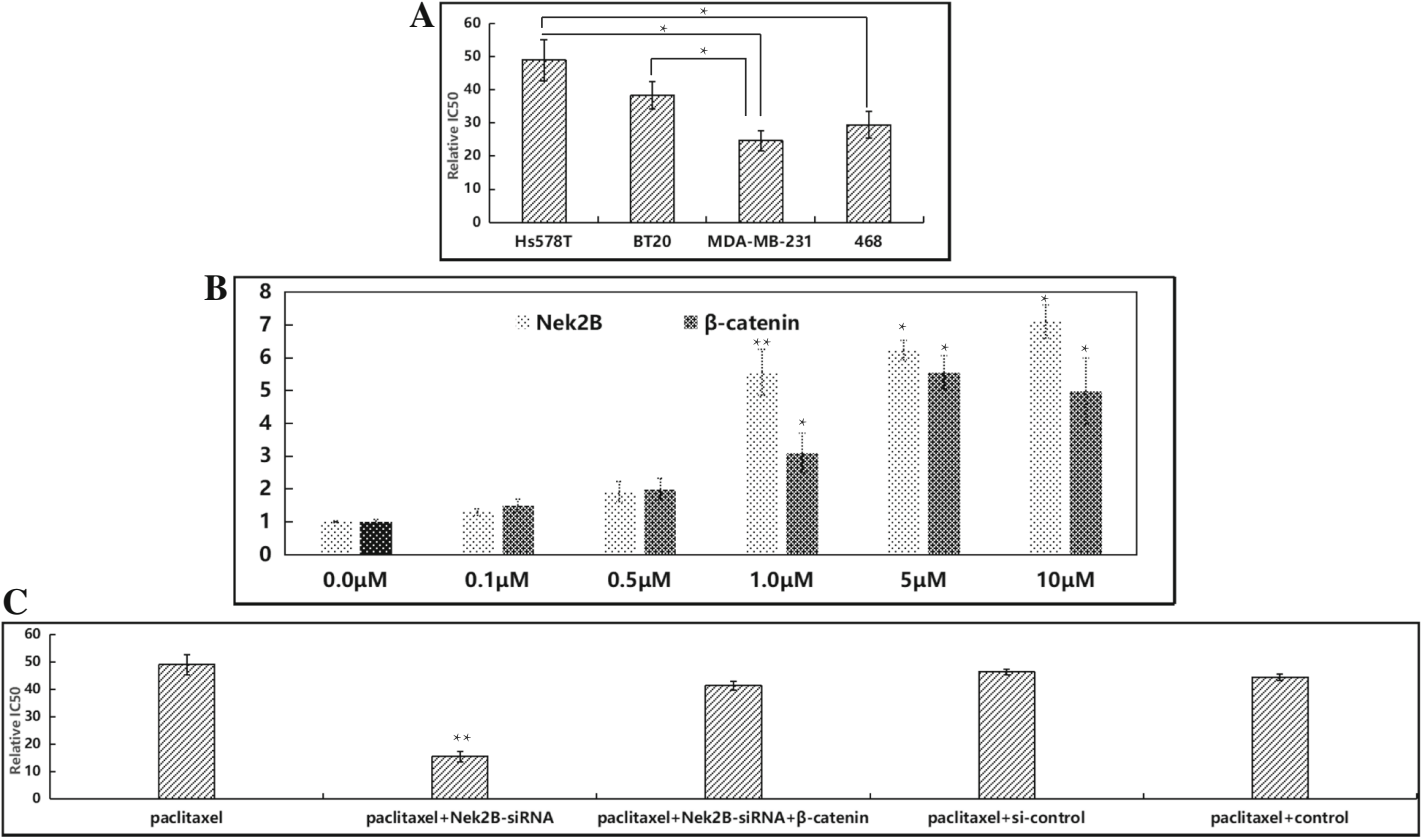
(**a**) CCK8 assay was used to detect the IC50 value of TNBC cell lines; (**b**) The Nek2B and β-catenin mRNA expression were detected by Q-RT-PCR after chemotherapy-sensitive cells MDA-MB-231 were treated with different concentrations of paclitaxel; (**c**) Nek2B-siRNA were transfected into drug-resistant cell Hs578T to test drug resistance reversal rate of Nek2B and β-catenin to paclitaxel. *P<0.05; **P<0.01

Human TNBC cell lines Hs578T, BT20, MDA-MB-231 and MDA-MB-468 were treated with paclitaxel at different concentrations for 72 h, and CCK8 was added. OD values were read by a microplate reader. As shown in Fig. 4a, IC50 of Hs578T, BT20, MDA-MB-231 and 468 cell lines were 48.98 ± 1.68 mol/L, 38.33 ± 4.16 mol/L, 24.66 ± 2.99 mol/L, and 29.45 ± 3.95 mol/L, respectively, with statistically significant differences (*P* < 0.05). Thus, Hs578T and BT20 cell lines were relatively resistant, while MDA-MB-231 and 468 cell lines were relatively sensitive. Further induction in chemotherapy-sensitive cells MDA-MB-231 with different concentrations of paclitaxel (0 M, 0.1 M, 0.5 M, 1 M, 5 M, 10 M) showed a concentration-dependent increase in Nek2B and β-catenin mRNA levels (Fig. 4b). We further transfected Nek2B-siRNA into drug-resistant cell Hs578T, showed that the drug resistance reversal rate of Nek2B to paclitaxel was 3.1 times (15.62 1.89 mol/L vs. 49.07 3.61 mol/L), and with the overexpression of β-catenin, the IC50 were increased (*P* < 0.05) (Fig. 4c), indicating that Nek2B-siRNA could improve the sensitivity of TNBC cell line to paclitaxel, suggesting the potential role of Nek2B and wnt/β-catenin pathways in the progression of drug resistance in TNBC cells.

### Nek2B and β-catenin expression status in TNBC cells

**Fig. 5:**
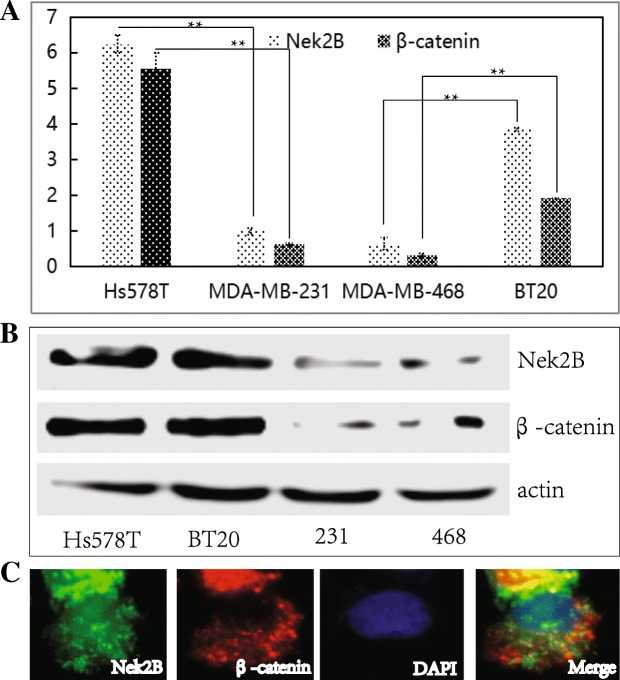
The mRNA (**a**) and protein (**b**) expression of Nek2B and β-catenin by Q-RT-PCR and Western blot in TNBC cells; Results are represented as mean ± S.E. **c** The expression pattern of Nek2B and β-catenin were showed by color images of immunofluorescence staining in Hs578T cells. *P<0.05; **P<0.01

We analysied the mRNA and protein expression of Nek2B and β-catenin by Q-RT-PCR (Fig. 5a) and Western-blot (Fig. 5b) in TNBC cells. It showed that the mRNA and protein expression level of Nek2B and β-catenin were higher in Hs578T and BT20 cells than in the MDA-MB-231 and MDA-MB-468 cells; Immunofluorescence double staining showed that Nek2B was located in the nucleus and cytoplasm, and β-catenin was mainly located in the cell membrane and cytoplasm (Fig. 5c). These results confirmed our preliminary hypothesis that there was a positive correlation between the expressions of Nek2B and β-catenin in TNBC cells.

### The targeted combination of Nek2B with β-catenin

**Fig. 6:**
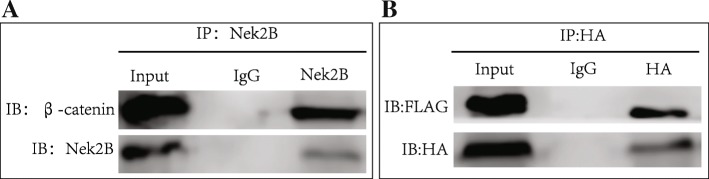
(**a**) CO-IP was used to detect the combination of endogenous Nek2B and β-catenin in Hs578T cells;(**b**) Hs578T cells were transfected of HA-β-catenin and FLAG-Nek2B, CO-IP was used to detect the combination of Nek2B and β-catenin

Immune protein coprecipitation (CO-IP) experiment showed that endogenous Nek2B and endogenous β-catenin exists in the form of compounds in Hs578T cells (Fig. 6a). At the same time, Hs578T cells were transfected with β-catenin of HA tag and Nek2B FLAG tag. FLAG-tag was used to precipitate Nek2B protein antibody, and Western blot showed the existence of β-catenin in the protein complexes (Fig. 6b). This further confirms that Nek2B can combine with β-catenin.

**Fig. 7:**
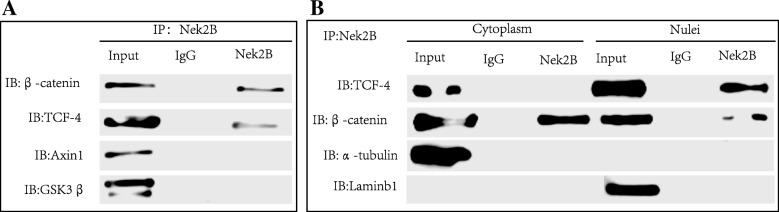
(**a**) CO-IP was used to detect whether Nek2B/β-catenin complex participate in the β-catenin phosphorylation and degradation in Hs578T cell; (**b**) Cytoplasm and nucleus of Hs578 cell line were detected by nucleoplasmic separation assay and CO-IP

We want to test whether the complex formed by Nek2B and β-catenin participate in the β-catenin phosphorylation and degradation using CO-IP in Hs578T cell. Nek2B antibody is used to immunoprecipitate β-catenin, TCF4, Axin l and GSK3β protein. As shown in Fig. 7a, Nek2B can be combined with β-catenin and TCF4, but not with Axin l and GSK3β. Cytoplasm and nucleus of Hs578 cell line were detected by nucleoplasmic separation assay and CO-IP, it showed that Nek2B, β-catenin and TCF4 can form complex in the nucleus (Fig. 7b). This suggests that the Nek2B and β-catenin complex directly enters the nucleus and forms a transcription complex with TCF4.

### Regulation of β-catenin by Nek2B in TNBC cell lines

**Fig. 8:**
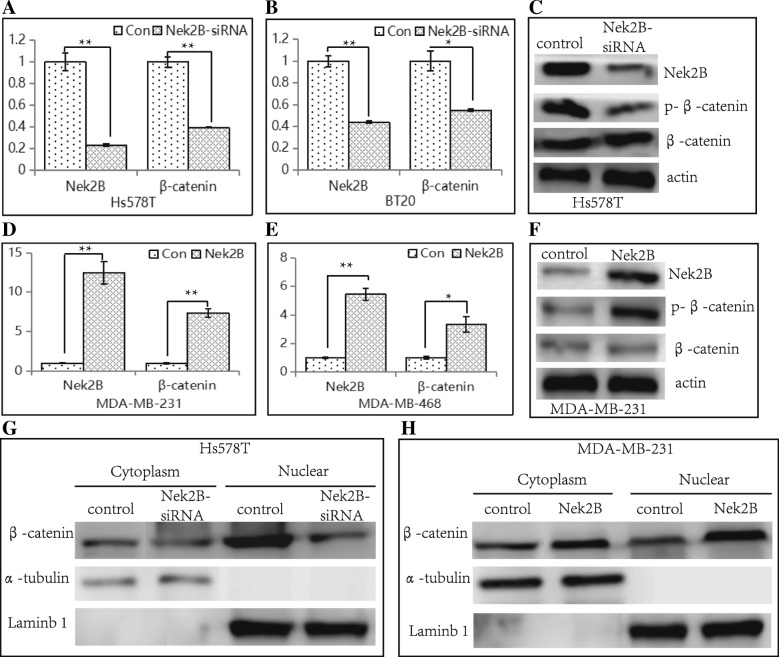
Hs578T (**a**) and BT20 (**b**) cells were transfected with Nek2B siRNA, Nek2B and β-catenin mRNA levels were analyzed by qRT-PCR; (**c**) Western blot analysis was used to detect the Nek2B and p-β-catenin protein expression in Hs578T cells after transfected with Nek2B siRNA. Actin is shown as loading control; MDA-MB-231(**d**) and MDA-MB-468 (**e**) cells were transfected with Nek2B plasmid, Nek2B and β-catenin mRNA levels were analyzed by qRT-PCR; (**f**) Western blot analysis was used to detect the Nek2B and p-β-catenin protein expression in MDA-MB-231 cells after transfected with Nek2B plasmid; Nucleoplasmic separation technique was used to detect the expression of β-catenin in nucleus and cytoplasm after Hs578T cells were transfected with Nek2B siRNA (**g**) and MDA-MB-231 cells were transfected with Nek2B plasmid (**h**). *P<0.05; **P<0.01

Nek2B-siRNA and Nek2B overexpression plasmids were transfected into Nek2B high expression cells and low expression cells, respectively. Data showed that the expression levels of both Nek2B and β-catenin mRNA were reduced in Hs578T(Fig. 8a) and BT20 cells (Fig. 8b) after Nek2B was interfered. Decreased expression of phosphorylated β-catenin (p-β-catenin) protein was found in Hs578T cells (Fig. 8c). The expression levels of both Nek2B and β-catenin mRNA were elevated in MDA-MB-231 (Fig. 8d) and MDA-MB-468 cells (Fig. 8e) after Nek2B was overexpressed. Increased expression of phosphorylated β-catenin (p-β-catenin) protein was found in MDA-MB-231 cells (Fig. 8f). Nucleoplasmic separation technique showed that after Nek2B was interfered in Hs578T cell line, the expression level of β-catenin in the nucleus decreased, but not in cytoplasm (Fig. 8g). When Nek2B was overexpressd in MDA-MB-231 cell line, the expression level of β-catenin in the nucleus increased, but not in cytoplasm (Fig. 8h). This indicates that Nek2B mainly regulates the expression of β-catenin in TNBC nucleus.

**Fig. 9:**
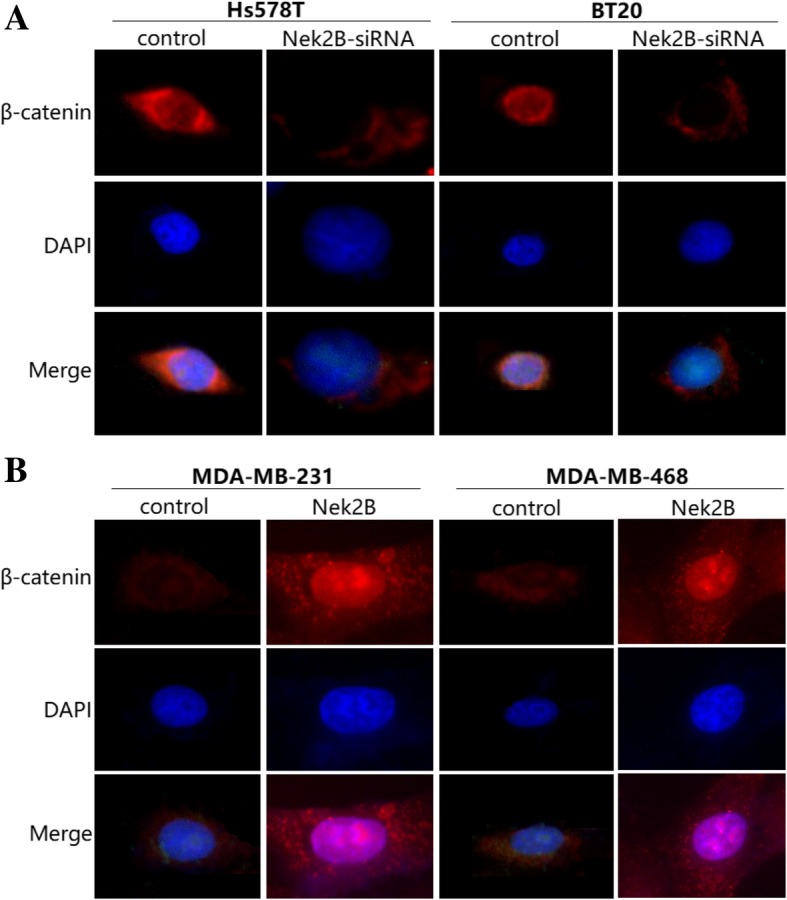
Immmunofluorescence was used to show the β-catenin expression after Hs578T cells and BT20 cells were transfected with Nek2B siRNA (**a**) and MDA-MB-231 cells and 468 cells were transfected with Nek2B plasmid (**b**)

Immmunofluorescence results showed that the nuclear penetration of β-catenin was weakened or even disappeared after Nek2B was interfered in Hs578T and BT20 cells (Fig. 9a), while the ability of nuclear penetration was obvious enhanced after Nek2B was overexpressed in MDA-MB-231 and 468 cells (Fig. 9b).

### The binding of Nek2B,β-catenin and LEF-1

**Fig. 10:**
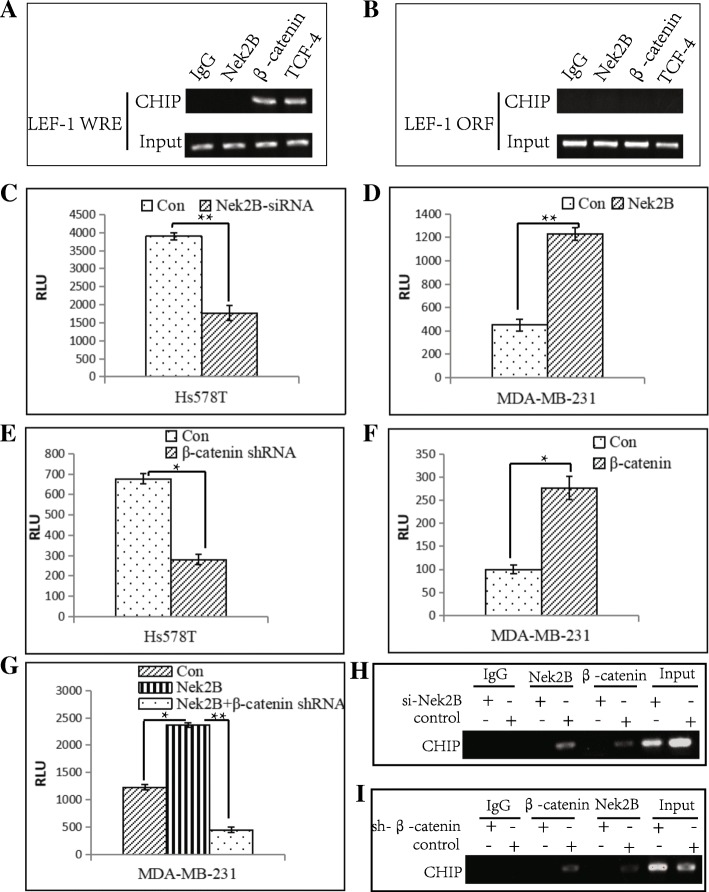
The relationship between Nek2B and LEF-l WRE (**a**) and LEF-l ORF (**b**) promoter was analysed using CHIP; (**c**)Hs578T cells were cotransfected with control or Nek2B-siRNA along with the pGL4-LEF-1-Prom-Luc reporter. Data points represent the mean ± S.D. of triplicate samples from two independent experiments. RLU: Relative Luciferase Units; (**d**) MDA-MB-231 cells were cotransfected with control or Nek2B along with the pGL4-LEF-1-Prom-Luc reporter; (**e**)Hs578T cells were cotransfected with control or β-catenin-shRNA along with the pGL4-LEF-1-Prom-Luc reporter; (**f**)MDA-MB-231 cells were cotransfected with control or β-catenin along with the pGL4-LEF-1-Prom-Luc reporter; (**g**) MDA-MB-231 cells were transfected with Nek2B alone or Nek2B and β-catenin shRNA together along with the pGL4-LEF-1-prom-Luc reporter. Repression of β-catenin could overcome the promotion of LEF-1 promoter activity by Nek2B; ChIP assays was used to detect the combination of β-catenin and LEF-1 promoter with the silence of Nek2B (**h**), and detect the binding of Nek2B to the LEF-1 promoter with the silence of β-catenin (**i**). *P<0.05; **P<0.01

To detect whether the Nek2B, β-catenin and TCF4 complex can bind to the LEF-l promoter region, we detected the binding ability of the complex with LEF-1 promoter in Hs578Tcell lines by chromosomal immunoprecipitation. The relationship between Nek2B and LEF-l promoter is shown in Fig. 10. It is found that Nek2B, β-catenin and TCF4 can be binded with the WRE functional area of LEF-1 promoter (Fig. 10a), while they can’t locate in the LEF-1 open reading framework (ORE) (Fig. 10b). To examine whether Nek2B affects the activity of LEF-1 promoter, Nek2B-siRNA or Nek2B plasmid were cotransfected with a luciferase reporter driven by the full-length promoter of the LEF-1 gene into Hs578T cells or MDA-MB-231 cells, respectively, and luciferase assays were performed. Down-regulation of Nek2B expression by Nek2B-siRNA decreased (Fig. 10c), whereas overexpression of Nek2B increased, the activation of the LEF-1 promoter (Fig. 10d). Down-regulation of β-catenin by β-catenin shRNA reduced LEF-1 promoter activity (Fig. 10e), whereas overexpression of β-catenin enhanced LEF-1 promoter activity (Fig. 10f). We next cotransfected Nek2B with β-catenin shRNA in MDA-MB-231 cells expressing a luciferase reporter driven by the full-length LEF-1 promoter. The results showed that Nek2B increased LEF-1 promoter activity, which was reversed by suppression of β-catenin (Fig. 10g). ChIP assays showed the combination of β-catenin and LEF-1 promoter disappears with the silence of Nek2B(Fig. 10h), similarly, after the interference of β-catenin, the binding of Nek2B to the LEF-1 promoter also disappeared (Fig. 10i). The above results show that Nek2B and β-catenin depend on each other to aggregate to LEF-1 promoter WRE region.

### Nek2B activates the wnt pathway

**Fig. 11:**
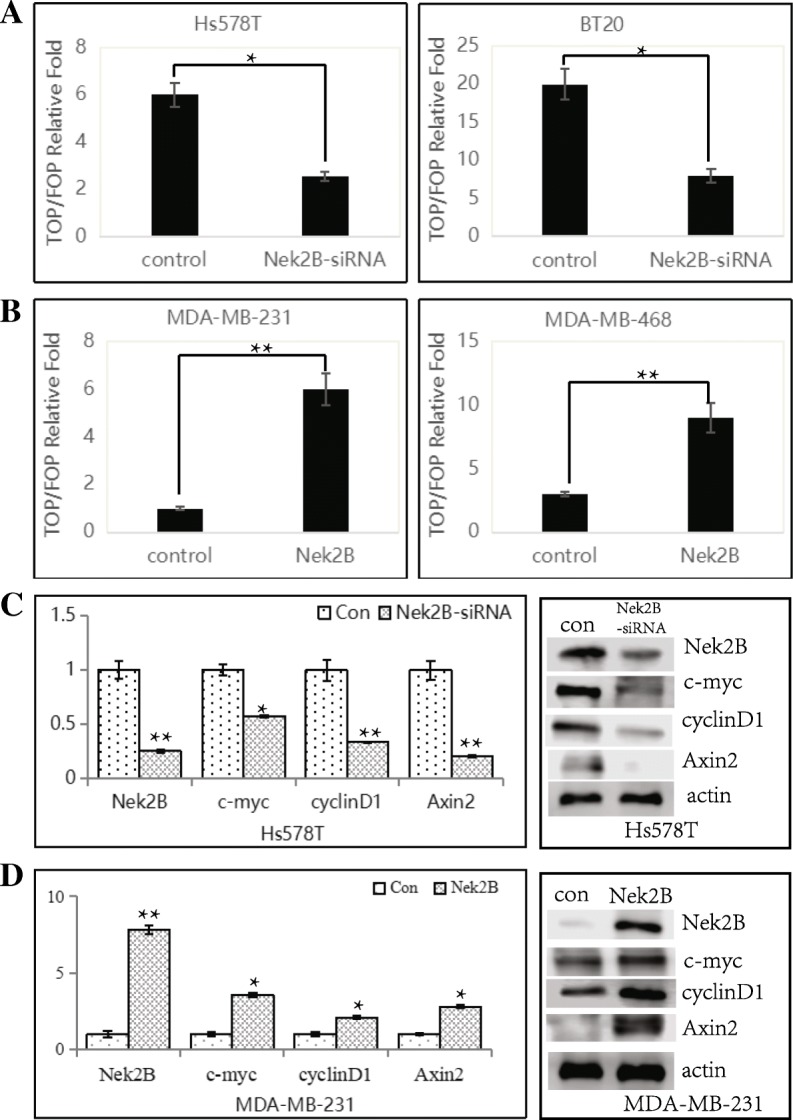
The activity of TOP/FOP was detected after Nek2B-siRNA or Nek2B plasmid were transfected to the cell lines Hs578T and BT20 (**a**) or MDA-MB-231 and 468 (**b**), respectively; PCR and westem blot assay were used to detect the expression of downstream target genes of wnt pathway after Nek2B-siRNA was transfected to Hs578T cells (**c**) and Nek2B plasmid were transfected to MDA-MB-231 cell (**d**). *P<0.05; **P<0.01

We previously found that Nek2B was closely related to the wnt signaling pathway using bioinformatics. Nek2B-siRNA or Nek2B plasmid were transfected to the Hs578T and BT20 or MDA-MB-231 and 468 cell lines, respectively. Down-regulation of Nek2B expression by Nek2B-siRNA decreased (Fig. 11a), whereas overexpression of Nek2B increased the activity of TOP/FOP (Fig. 11b). These results further confirm that Nek2B can activate wnt signaling pathway. To detect whether Nek2B affects the expression of downstream target genes of wnt pathway, PCR and westem blot assay were used to detect the expression of c-myc, cyclinDl and Axin2 of Hs578T and MDA-MB-231 cell lines. After interfering with Nek2B in Hs578T cell lines, the expression of c-myc, cyclinDl and Axin2 were decreased significantly (Fig. 11c). It was found that the expression of c-myc, cyclinDl and Axin2 were upregulated with the overexpression of Nek2B(Fig. 11d), indicating that Nek2B not only combines with β-catenin and TCF4 to form complex, and bind to LEF-1 promoter, but also regulates the expression of wnt downstream target genes.

### Nek2B activates the wnt pathway by stabilizing β-catenin

**Fig. 12:**
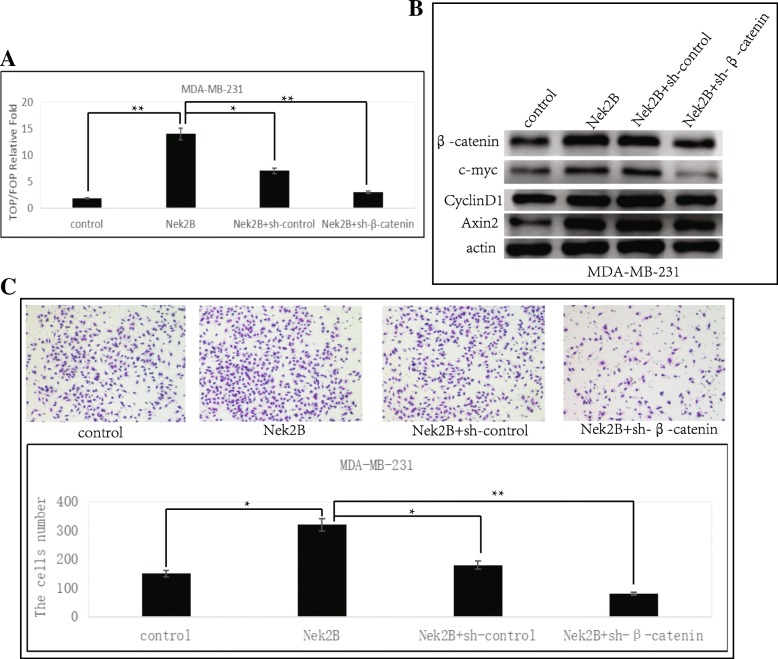
Nek2B activates the wnt pathway by stabilizing β-catenin. Control, Nek2B, Nek2B + sh-control or Nek2B+ sh-β-catenin plasmids were transfected into MDA-MB-231 cell lines, and the expression levels of TOP/FOP reporting system (**a**) and the downstream target genes c-myc, cyclinDl and Axin2 of wnt signaling pathway were detected (**b**) respectively;(**c**) Transwell cell invasion experiment was used to verify the changes in the invasion ability of MDA-MB-231 cell line after transfection of control, Nek2B, Nek2B + sh-control or Nek2B + sh-β-catenin plasmids. *P<0.05; **P<0.01

To determine the association between Nek2B activates wnt signaling pathway and β-catenin, control, Nek2B, Nek2B + sh-control or Nek2B + sh-β-catenin plasmids were transfected into MDA-MB-231 cell lines, the expression levels of TOP/FOP reporting system (Fig. 12a) and the downstream target genes c-myc, cyclinDl and Axin2 of wnt signaling pathway were detected respectively (Fig. 12b). It was found that after overexpression of Nek2B, the activity of wnt signaling pathway increased, and with the knockout of β-catenin, not only the activity of wnt signaling pathway was significantly down-regulated, but also the protein expression levels of c-myc, cyclinDl and Axin2 were decreased. Then we used transwell cell invasion experiment to verify the changes in the invasion ability of MDA-MB-231 cell line after transfection of control, Nek2B, Nek2B + sh-control or Nek2B + sh-β-catenin plasmids (Fig. 12c). It was found that the invasion ability of MDA-MB-231 cell line was significantly increased after overexpression of Nek2B, while decreased with the knockout of β-catenin. The statistical results of the invasion experiment clearly suggest that Nek2B and β-catenin co-promote tumor cell invasion and metastasis.

### Combination treatment using Nek2B siRNA and paclitaxel in nude mice

**Fig. 13:**
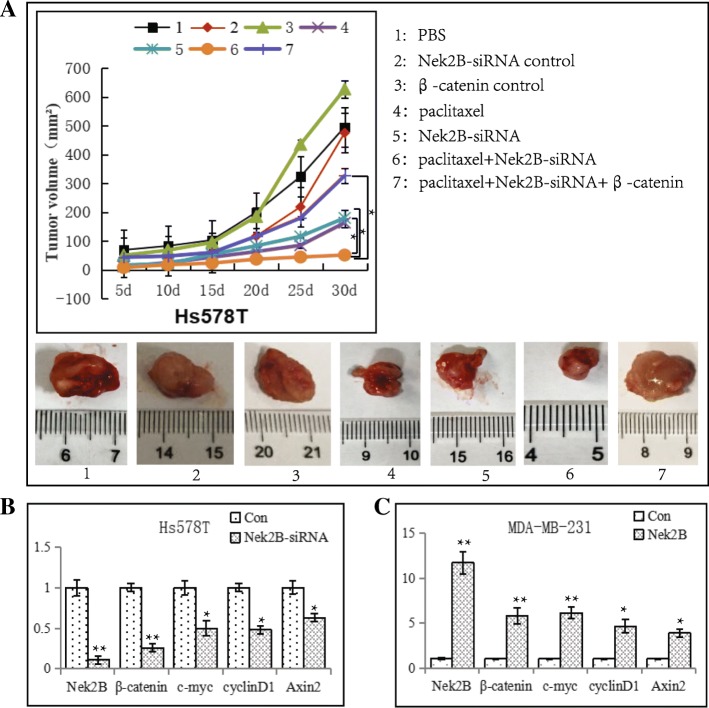
In vivo experiments (**a**) The nude mice were divided into five groups (1–7) by different treatment, 1: PBS, 2: Nek2B-siRNA control, 3:β-catenin control, 4:paclitaxel, 5: Nek2B siRNA, 6: Nek2B siRNA combining with paclitaxel, 7: Nek2B siRNA and paclitaxel combining with β-catenin. Total RNA extracts from the tumors which were transfacted with Nek2B-siRNA in Hs578T cells (**b**) and Nek2B plasmid in MDA-MB-231 cells (**c**) were examined for expression levels of Nek2B, β-catenin and downstream target genes. *P<0.05; **P<0.01

To further detect the effect of Nek2B and β-catenin in TNBC chemotherapy resiatance, we performed a combination treatment using Nek2B siRNA and paclitaxel which disrupted the therapeutic targets of spindle poisons in nude mouse model. As shown in Fig. 13a, the tumor volume in Nek2B siRNA combining with paclitaxel group was decreased compared with Nek2B siRNA group (*P* < 0.01) and paclitaxel group (*P* < 0.05), respectively. With the overexpression of β-catenin, the tumor volume were increased. The data demonstrated that combination treatment using Nek2B siRNA and paclitaxel may suppress TNBC tumor growth rate more efficiently than paclitaxel alone or Nek2B siRNA alone, indicating the potential role of Nek2B and wnt/β-catenin pathways in the progression of drug resistance. Total RNA extracts from the tumors were examined for expression levels of Nek2B, β-catenin and downstream target genes(c-myc, cyclinD1, Axin2) of wnt pathway. The results indicated that Nek2B can regulate the expression of β-catenin and wnt downstream target genes in vivo (Fig. 13b, c).

## Discussion

Nek2B plays an established role in centrosome separation, and a number of centrosomal substrates have been identified [17, 18]. Nek2B has also been shown to negatively regulate primary cilia assembly, since ablation of the kinase led to an increase in cilia assembly and a delay in disassembly [19]. Despite these observations, mechanistic insights and substrates were lacking. Here we identify Nek2B and β-catenin were co-expressed in TNBC and correlated with the patients prognosis, and β-catenin may be a major substrate of Nek2B. To our knowledge, this is the first report demonstrating the role of Nek2B and β-catenin co-expression status in TNBC.

Chemotherapy remains the mainstay for the treatment of TNBC due to lack of targeted therapies [20]. However, despite initial chemosensitivity, metastatic relapse paradoxically appears to occur at higher rates for TNBC [21]. In the present study, patients with positive Nek2B expression developed drug resistance to paclitaxel-containing neoadjuvant chemotherapy. We also found chemothearpy-resistant TNBC cells showed higher expression of Nek2B and β-catenin. This is consistent with Misra et al. [22] and TIEN et al. [23] findings, they put forward that Nek2 stimulates the expression of ABC transporters by activation of AKT and its downstream targets PIM1 and NF-kB, resulting in drug resistance of cancer cells. We also found that Nek2B-siRNA could improve the sensitivity of TNBC cell line to paclitaxel, and β-catenin can increase drug resistance rate of Nek2B-siRNA to paclitaxel, suggesting the potential role of Nek2B and wnt/β-catenin pathways in the development and progression of drug resistance in TNBC cells. Although it has been previously shown that Nek2 depletion affected the kinetochores-microtubule attachment, inducing a spindle checkpoint imbalance and abnormal clustering of kinetochore components, resulting in increased sensitivity of cells to the microtubule targeting anticancer drug paclitaxel [8] in contrast, Spallarossa et al. [24] described that the increase in cell sensitivity of Nek2-depleted TNBC cells in combination with doxorubicin may be due to the down-regulation of TRF1 and the checkpoint kinase, Chk2, inducing chromosomal abnormalities and altering the cell cycle. Knockdown of β-catenin in TNBC cells significantly decreased cell migration and made TNBC cells more sensitive to chemotherapeutic drugs like cisplatin and doxorubicin [25]. Zhou et al. [26] pointed that knock-down of β-catenin abrogates Nek2-induced colony formation and drug resistance indicating that β-catenin plays indeed an important role in Nek2 function. Our study showed that the patient group with positive co-expression pattern of Nek2B and β-catenin had the worst DFS in TNBC patients, and Nek2B and β-catenin co-promote tumor cell invasion and metastasis. The association of Nek2B or β-catenin with Ki67 suggests that the decreased outcomes are related to increased proliferation in these tumours.

**Fig. 14:**
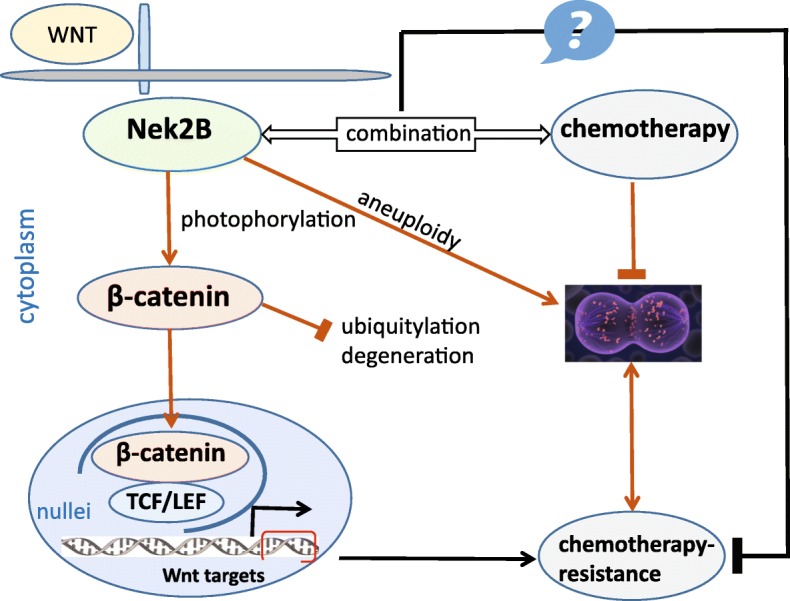
A hypothesis working model for Nek2B-mediated chemotheropy-resistant mechanism in TNBC. In the classical wnt signaling pathway, the targeted combination of Nek2B and β-catenin can promote the entry of β-catenin into the nucleus and activate the transcription factor TCF/LEF, and then activate the downstream target genes of the wnt pathway and promote drug resistance of TNBC

As the literatures reported by Zhang et al. [27] and Seung et al. [28], our study showed that Nek2B can combine with β-catenin and directly enters the nucleus and forms a transcription complex with TCF4, and Nek2B mainly regulates the expression of β-catenin in TNBC nucleus. Wnt signaling plays a key role in many tissues and has been implicated in several cancers. Activation of wnt signaling induces cancer cell survival and drug resistance [29]. When cytoplasmic β-catenin is stabilized, it translocates into the nucleus, where it interacts with a family of T-cell factor/lymphocyte enhancer factor (TCF/LEF) transcription factors to drive cell proliferation by direct induction of cell cycle regulators, such as c-Myc and cyclin D1 [29]. The TCF/LEF transcription factors appear to be the major nuclear targets of β-catenin. The TCF/β-catenin complex also regulates participating in drug resistance [30]. By chromosome immunoprecipitation (CHIP) assay, we found that Nek2B and β-catenin can co-locate on the TCF/LEFl promoter WRE sequence. And when Nek2B is knocked out, the combination of β-catenin with the TCF4/LEF-l promoter also disappears. Correspondingly,the binding of Nek2B with TCF4/LEF-1 also disappears with the knockout of β-catenin. These phenomena suggest that Nek2B can not only promote β-catenin aggregates into the nucleus, but also mediate the formation of Nek2B-catenin-TCF/LEF transcriptional compound. Nek2B can not only increase the activity of wnt signaling pathway, but also regulate the expression of wnt downstream target genes. Significantly, recent studies also support important roles of both Nek2B and canonical wnt/β-catenin signaling in breast cancer [18, 31]. Based on these findings, we believe that our study establishes a novel link between Nek2B and wnt pathway activation in TNBC and shows mechanistically that Nek2B positively regulates multiple genes required for wnt signaling. Overall, these findings indicate that wnt synthesis and signaling are driving at least a subset of TNBC, in particular those tumors with high Nek2B levels, and that this subset of TNBC may be responsive to wnt synthesis inhibitors or other wnt pathway antagonists that are now entering the clinic. According to the results, we speculate a new layer of Nek2B-mediated chemotherapy-resistant in TNBC (Fig. 14).

Limitations of the current study’s findings must be made. There is no validated approach for the assessment of Nek2B or β-catenin by immunohistochemistry, and we elected to use methods by Lee et al. [32]. On the other hand, our study was restricted to a solitary center and a multicenter study is needed to confirm our results. However, more study is needed to elucidate their relationship with Nek2 and the implications for cancer development.

## Conclusion

In conclusion, our study showed, for the first time, that Nek2B can bind to β-catenin and the co-expression correlated with TNBC patients poor prognosis. It appears that Nek2B and β-catenin might synergize to promote chemotherapy resistance. Further studies should include TNBC specimens and cells to determine if Nek2 and β-catenin might synergize to promote chemotherapy resistance.

## Abbreviations

CHIP: Chromatin immune-precipitation
CO-IP: Co-immunoprecipitation
DFS: Disease-free survival
GEO: Gene Expression Omnibus
OS: Overall survival
pCR: Pathological complete response
PD: Disease progression
PR: Partial response
SD: Stable condition
siRNA: Small interfering RNA
TCF/LEF: T-cell factor/lymphocyte enhancer factor
TNBC: Triple-negative breast cancer
